# Livestock owners’ worry and fear of tick-borne diseases

**DOI:** 10.1186/s13071-020-04162-7

**Published:** 2020-06-30

**Authors:** Maria Johansson, Atle Mysterud, Anders Flykt

**Affiliations:** 1 Environmental Psychology, Department of Architecture and the Built Environment, Lund University, Lund, Sweden; 2 Centre for Ecological and Evolutionary Synthesis (CEES), Department of Biosciences, University of Oslo, Oslo, Norway; 3 Department of Psychology, Mid Sweden University, 831 25 Östersund, Sweden

**Keywords:** Livestock owners, Tick-borne disease, Fear, Coping potential

## Abstract

**Background:**

Recent global changes have led to an increase in distribution of ticks towards higher elevation and latitude in Europe and livestock are at increasing risk of contracting tick-borne diseases, but psychological aspects of how this affects human well-being are rarely assessed. Departing from the theory on emotional appraisal coming from psychology, this study investigates which factors that modulate worry and fear associated with the presence of ticks among livestock owners of sheep and/or cattle.

**Methods:**

Survey data from 775 livestock owners in Norway were analysed by hierarchical multiple regression analysis with an index of fear of tick-borne diseases among livestock as the outcome variable.

**Results:**

Twenty-nine per cent of the livestock owners reported worry and fear of tick-borne diseases among their livestock. The model explained 35% of the variance in worry and fear. There was a weak association between estimated incidences of tick-borne diseases in livestock and livestock owners’ worry and fear. Whereas previous personal experience of ticks and tick-borne diseases in livestock, and the livestock owners’ appraisals of the situation were more strongly associated with relatively stronger feelings of worry and fear.

**Conclusions:**

Livestock owners’ worry and fear of tick-borne diseases in livestock can partly be understood as their appraisals of perceived personal relevance of the presence of ticks, its potential negative implications for their daily life at large, and what potential they have to cope by different strategies to adapt or adjust to the situation.
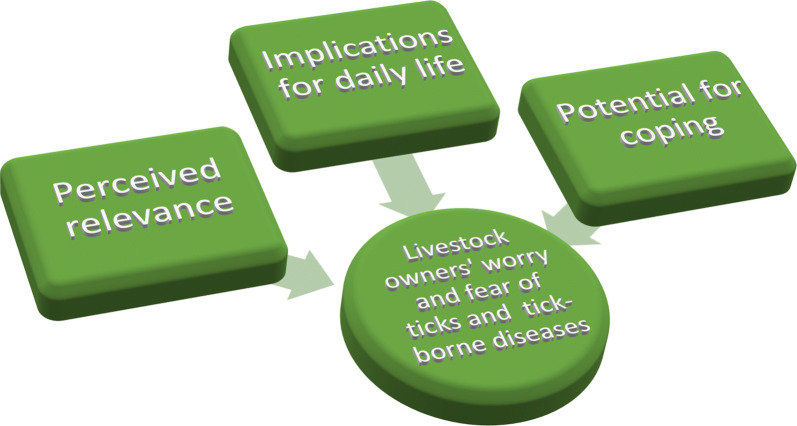

## Background

Recent global changes have led to an increase in distribution of ticks towards higher elevation and latitude in Europe [1] and in North America [2], and both humans and livestock are at increasing risk of contracting diseases vectored by ticks [3]. The generalist ticks *Ixodes ricinus* in Europe, *I. scapularis* and *I. pacificus* in North America and *I. persulcatus* in parts of Europe and across Asia transmit a variety of pathogens [4], which can cause what is usually referred to as ‘tick-borne’ diseases. Most common among the tick-borne pathogens are bacteria from the *Borrelia burgdorferi* (*sensu lato*) complex causing Lyme borreliosis in humans [5]. Another common tick-borne pathogen is *Anaplasma phagocytophilum* causing livestock fever (anaplasmosis) in cattle and sheep [6], and also the piroplazmid *Babesia divergens* regularly cause babesiosis among livestock in Europe [7]. A lot of effort has been devoted to estimate the disease hazard, which is defined as the density of infected nymphal ticks in a given ecosystem [8, 9]. Disease hazard is used to assess how the public perceive the associated risks of developing tick-borne diseases in humans [10], and identify drivers of adoption of protective behavior [11–13].

In research based on behavioral theories the concept of self-efficacy stands out as a critical component in the adoption of protective behavior, meaning the belief the person has about his or her ability to perform the behavior in question [14–16]. We know that groups spending time outside/in nature such as foresters, wildlife managers and scouts are at extra-risk for contracting Lyme borreliosis due to increased exposure [17], and may need special attention to their situation to reduce risks of tick-borne diseases [18]. Those having high likelihood of being exposed to ticks have increased worry and fear for their personal health [19]. One group that so far has received little attention in the literature of fear of tick-borne diseases is farmers, who are not only exposed themselves, but also their livestock. Tick-borne diseases among livestock may potentially increase workload in terms of adopting preventive measures for their livestock and financial stress due to loss of animals among livestock owners as well as constituting a source of psycho-social stress impacting on perceived quality of life. In this study, we focus upon how farmers respond to presence of ticks and tick-borne diseases among their livestock, and in particular to what extent they express worry and fear.

In psychology worry and fear could be regarded as different intensity of the same emotion [20]. The worry and fear that the livestock farmers could experience from the perceived likelihood that their animals will be infested with ticks could be addressed in different ways. Psychological theory can disentangle what the core of the cause to this worry and fear is. A model like the Component Process Model of emotional appraisal [21], states that an appraisal process results in emotions and can be understood as four basic subsequent and interconnected steps. In the appraisal process, the thought of the probability that one’s animals will be exposed to ticks and may get a tick-borne disease is first considered with respect to its relevance in relation to one’s goals (e.g. having healthy animals, a large production, a good net income). That is, if the relevance is high, the likelihood for experience fear of tick-borne diseases in livestock would be higher than if relevance is low. Secondly, if relevant, the individual considers the implications of the possible event, e.g. possible implications for perceived quality of life such as workload, reduced opportunities for recreation or financial stress. The more negative the implications are, the more they would contribute to worry and fear. Thirdly, if these implications are thought of as negative, for example an obstruction to reach the goal, the individual possibilities to cope with the situation are considered (i.e. the coping potential). The coping potential is about the individual’s ability to handle a situation. Strategies to handle situations may range from emotion-based strategies, such as becoming angry, feeling hopeless and giving up, to more problem-solving based strategies for example finding information about how to counteract the presence of ticks, or take the necessary measures to prevent animals getting sick (the latter similar to self-efficacy). Social trust in representatives of local and national authorities as well as representative stakeholder organizations might be supportive for the coping potential [22]. Eventually an evaluation about how the situation and the appraisal of the situation are congruent with the personal and the societal norms, for example those communicated by authorities and stakeholders, is made. The less congruent, the more likely this appraisal contributes to negative emotions.

In Denmark, Finland, Norway and Sweden people are worried about tick-borne diseases among humans and to a certain extent also adopt protective behavior for themselves and their pet animals [10, 23, 24]. There is also anecdotal evidence from stakeholders that emergence of tick-borne diseases among livestock would cause worry and fear among farmers, but we lack an understanding of such worry and fear. Departing from theory on emotional appraisal, we analyze which factors that modulate worry and fear associated with the presence of ticks among livestock owners (sheep and cattle) in Norway. The aims are to understand livestock owners’ emotional appraisal of densities of ticks and levels of tick-borne disease incidences related to livestock, and to test if the livestock owners’ emotional appraisal is associated with their reported worry and fear. More specifically we ask:

- Q1: Is the increasing presence of ticks in the environment relevant to livestock owners’ way of handling their husbandry?
- Q2: If so, what are the livestock owners’ perceived implications of the presence of ticks in the environment and tick-borne diseases in livestock?
- Q3: How do livestock owners cope with the new situation if they are perceived to be constrained by the presence of ticks in the environment and tick-borne diseases in livestock?
- Q4: Is the solution(s) arrived at considered acceptable or not by livestock owners with regard to perceived norms?
- Q5: Is the emotional appraisals similar across livestock owner groups, regardless of if the livestock is sheep, cattle or both?

It is expected that livestock owners who find the presence of ticks in the environment relevant to their way of handling their husbandry, the consequences to primarily be perceived to have negative implications, with low perceived possibilities to cope with the situation, and ways of handling the situation perceived as incongruent with norms are more likely to express worry and fear for their livestock.

## Methods

### Sample

The participants included a total of 775 (corresponding to a response rate of 27%) livestock owners with cattle and/or sheep (and in a few cases goats, *n* = 17) in areas with presence of ticks in the environment in Norway (18% females 30–77 years-old, mean (M) = 53 years, 82% males, 24–92 years-old, M = 56 years). The participants can be divided into three sub-samples based on their livestock (cattle only (sample size, *n* = 144), sheep only (*n* = 459), and cattle and sheep (*n* = 172). The characteristics of their farming in the three sub-samples are presented in Table 1.

**Table 1:** Characteristics of the three sub-samples and their farming practices in Norway

Variables	Cattle *n* = 144	Sheep *n* = 459	Cattle and sheep *n* = 172
Years on farm, M ± SD (range)	22 ± 11 (1–45)	26 ± 14 (1–70)	24 ± 12 (1–54)
Area (ha)			
0.0–4.9	3%	37%	7%
5.0–9.9	23%	36%	27%
10.0–19.9	40%	21%	36%
20.0–29.9	21%	4%	17%
30.0–49.9	9%	1%	9%
> 50.0	4%	1%	4%
Percentage of income, M ± SD (range)	82 ± 25 (5–100)	29 ± 25 (0–100)	19 ± 18 (0–90)
Winter stock, M ± SD (range)			
Cattle	58 ± 37 (6–230)		50 ± 37 (2–220)
Sheep		67 ± 49 (4–400)	45 ± 36 (0–272)
Production type			
Milk	97%	65%	60%
Meat	75%	53%	52%
Live animal sales	44%	69%	56%
Others	3%	70%	51%
Grazing cattle weeks, M ± SD			
Infield/pastures	13.9 ± 8.6		10.2 ± 8.4
Outfield mountain summer	9.7 ± 8.9		5.8 ± 7.7
Outfield forest/lowland summer	13.6 ± 9.7		8.8 ± 14.0
Grazing sheep weeks, M ± SD			
Infield/pastures in spring		4.9 ± 5.0	4.8 ± 4.4
Outfield mountain summer		11.3 ± 6.1	12.3 ± 5.9
Outfield forest/lowland summer		4.1 ± 6.6	5.3 ± 7.8
Infield/pastures in autumn		5.4 ± 5.2	5.8 ± 4.6

*Abbreviations*: M, mean; SD, standard deviation

### Study area

**Fig. 1:**
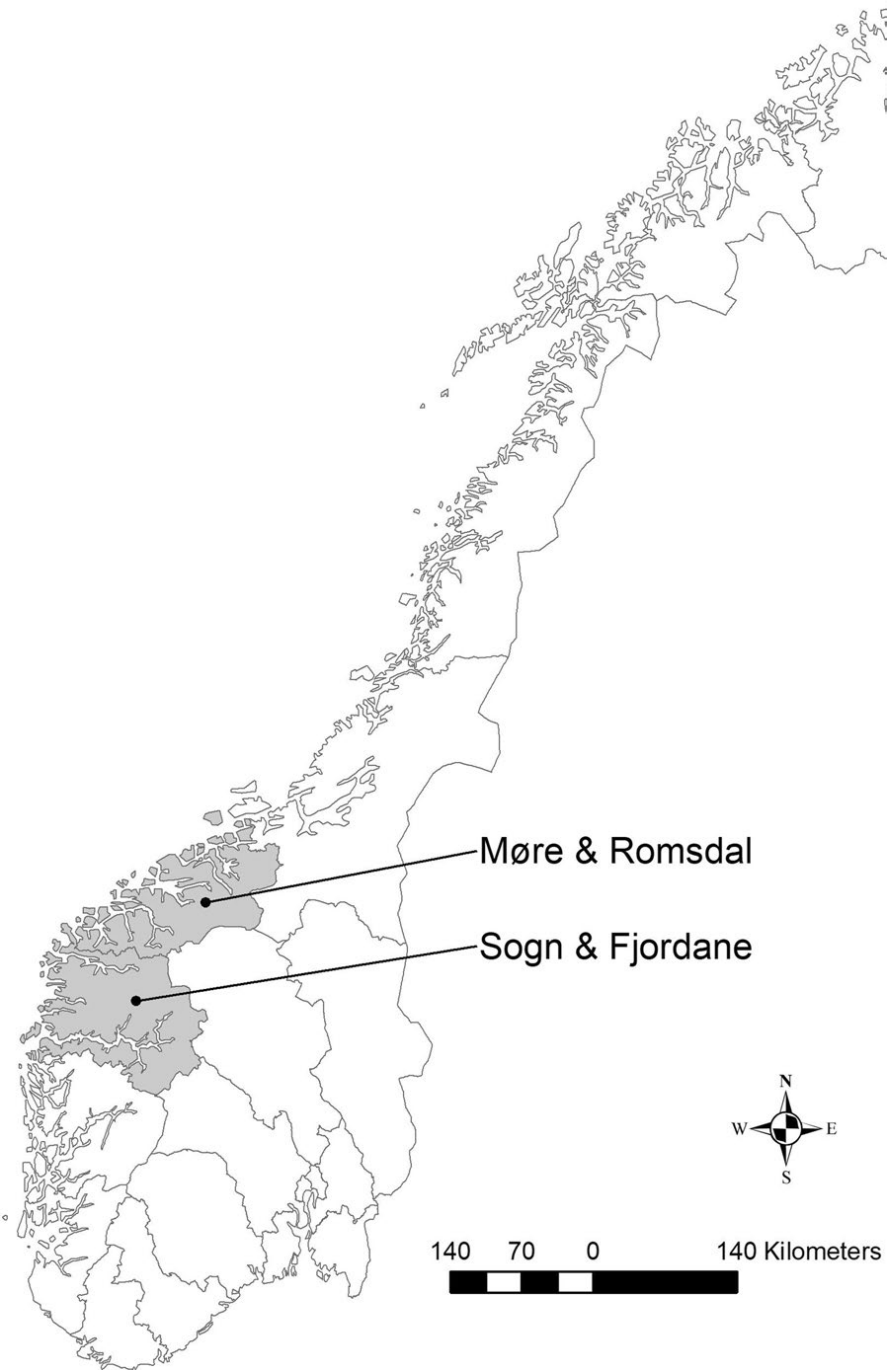
The study areas in Norway

The study area comprises all municipalities in the two counties Sogn & Fjordane and Møre & Romsdal in Norway (Fig. 1). The topography is characterized by high mountains interspersed with valleys and fjords. The climate is oceanic with temperature and precipitation declining from coast to inland. The vegetation is within the boreonemoral zone and dominated by Scots pine (*Pinus sylvestris*), alder (*Alnus incana*), birch (*Betula* spp.), and scattered stands of planted Norway spruce (*Picea abies*) [25]. The density of ticks is high up to roughly 200 m above sea level, but ticks are present up to some 500 m above sea level [25, 26]. Prevalence of Lyme borreliosis among humans is high, and anaplasmosis in sheep and anaplasmosis and babesiosis in cattle have both increased in prevalence and expanded geographically in both regions [3]. There are no resident large predators in the region, while red fox (*Vulpes vulpes*) are present and occasionally prey on lambs.

### Instruments

The questionnaire covered seven topics (A–G):

(A) Background questions: covering information about farming and livestock, place attachment and connection with nature, and experience of disease among the animals caused by ticks.
(B) Worry and fear of tick bites and tick-borne diseases in livestock was assessed by three items treated as an averaged index: (i) How strong worry/fear do you feel that your livestock will get tick bites during the next grazing season? (ii) How strong worry/fear do you feel that a tick bite could result in babesiosis? and (iii) How strong worry/fear do you feel that a tick bite could result in anaplasmosis? (internal reliability Chronbach’s α = 0.90). In addition, questions were asked about the how strong worry/fear they felt about attacks from large carnivores, and how serious the perceived it to be bitten by ticks themselves, to get Lyme borreliosis respectively tick-borne encephalitis (TBE). The response format was 1 = no worry/fear to 11 = very strong worry/fear, respectively 1 = not serious to 11 = very serious.
(C) Relevance was captured by two overarching questions covering six items: Have you noticed some effect of the presence of ticks in the Norwegian nature in relation to…?: (i) your everyday life; (ii) possibilities for recreational activities in the nature; (iii) possibilities for farming livestock; (iv) How do you experience the effects of tick bites that results in illness in the livestock? the effects: (v) have high relevance for me keeping livestock; (vi) and extensive for my livestock keeping (response format Likert scale: definitively not = 1 to agree = 5, Cronbach’s α = 0.88).
(D) Implication was assessed as the impact of the presence of tick-borne disease in livestock on subjective quality of life as measured in terms of 22 items [27], translated into Norwegian and slightly adapted to the Scandinavian context [28]. Responses were given in Likert scales: 1 = very negative impact to 5 = very positive impact.
(E) Coping potential was assessed by 15 items specifically developed for the present study. The items covered a diverse set of potential strategies identified together with representatives from the stakeholder organisations of cattle (TINE) and sheep (NSG) farmers (Table 2). Responses were given in Likert scales: 1 = definitely not to 5 = to the highest degree. In addition, the respondents were asked to elaborate on what strategies they use today to prevent tick-bites and tick-borne disease in livestock.

**Table 2:** Summary of exploratory factor analysis results (*n* = 677) with mean values and standard deviations of the items

Item	Distribution		Rotated factor loadings (varimax)		
	Mean	SD	Emotion	Action	Information-seeking
Search for information *via* adminstrations	3.75	1.12	0.23	0.09	**0.63**
Search for information on the internet	3.91	1.05	0.16	0.28	**0.62**
Contact stakeholder organisations	2.91	1.13	0.30	0.15	**0.64**
Feel worry/fear about how to handle the situation	3.27	1.16	**0.62**	0.09	0.43
Feel worry/fear for being accused for poor animal keeping	2.69	1.31	**0.74**	0.07	0.16
Become angry over the situation	2.78	1.18	**0.70**	0.07	0.17
Consider resigning from animal farming	2.31	1.25	**0.73**	0.03	0.06
Avoid talking about the problem with others	1.56	0.93	**0.46**	.014	**− 0.50**
Increase surveillance of the animals	4.28	0.82	0.01	**0.45**	0.40
Move foraging area	2.63	1.29	0.07	**0.77**	0.07
Keep animals indoors more frequently	2.44	1.24	0.20	**0.71**	**− **0.02
Clean the grazing area to reduce encroachment (removing bushes)	3.65	1.12	**− **0.16	**0.64**	0.20
Make changes in production	2.48	1.14	0.33	**0.53**	0.18
Eigenvalue			2.50	2.13	1.93
Percent of variance			19.25	**16.41**	**14.92**
Cronbach’s alpha			0.70	0.66	0.65

*Note*: Values above 0.45 indicated in bold
(F) Moreover, an established set of questions to assess social trust was included [29, 30]. The scale includes three items, I have trust in representatives from (i) state administrations, (ii) stakeholder organisations for livestock farming, and (iii) researchers focusing upon farming (Cronbach’s α = 0.78, response format: 1 = definitely not to 5 = to a high degree). In addition, they received open-ended questions on who they received support from and what kind of support they would have wished for.
(G) Norm compatibility was assessed by four items: (i) present preventive actions are effective; (ii) preventive actions are doubtful to use in my livestock farming (reversed); (iii) present actions are in line with what I consider good/quality livestock farming; (iv) the view of the community on tick bites makes it difficult to use preventive actions (reversed) (Cronbach’s α = 0.65).

### Procedure and analysis

Participants with cattle were recruited *via* TINE (Norwayʼs largest producer, distributor and exporter of dairy products with 11,400 members; owners and 9000 cooperative farms). An e-mail with a link to the online survey was sent by TINE to all 1275 members in the counties of Sogn & Fjordane and Møre & Romsdal. Participants with sheep and goat were recruited *via* Norsk Sau og Geit (NSG) a member organization for all sheep and goat farmers in Norway. All members in the geographical areas (i.e. districts) of Sogn & Fjordane and Møre & Romsdal who had registered an e-mail address received a link from NSG to the online survey (771 members) and those without an e-mail address (830 members) received a questionnaire by post. A reminder was sent either by e-mail or post approximately two weeks later. A joint database comprising protocols from the online survey and the post-survey was set up. Statistical analyses were performed in SPSS Statistics 22. Missing values were replaced by mean values and indices for the theoretical concepts investigated were constructed. Sub-dimensions of the concept coping potential were identified by means of a systematic exploratory factor analysis. Differences in assessments of the four appraisal dimensions between the three groups of livestock owners were tested with analysis of variance (ANOVA) and Bonferroni *post-hoc* tests to further identify which groups differed. The partial eta-squared (η_p_^2^) was used as a measure of effect size and reported for statistically significant results. Hierarchical multiple linear regression was used to test the possibility of predicting worry and fear for tick-borne disease with personal experience and the appraisal dimensions. All regressions were adjusted for potential moderators, i.e. livestock owner group. In all tests, *P*-values > 0.05 were considered to indicate statistical significance.

## Results

### Livestock owners’ worry and fear of tick-bites and tick-borne diseases

In general, the livestock owners’ worry and fear of tick-bites and tick-borne diseases among their livestock was low with a mean value on the lower part of the 11-graded scale (mean, M = 4.84, standard deviation, SD = 2.85). There was no significant difference between the three livestock owner groups, *F*_(2, 772)_ = 1.98, *P* = 0.138. Still one third of the respondents (29%) reported a mean value of 7 or above on the scale suggesting that they are clearly worried about the situation. As a reference, their mean value of worry and fear of attacks from large carnivores was M = 5.61, SD = 3.50, and their mean value on an index of perceived seriousness of personally getting tick-bites, Lyme borreliosis or TBE was M = 7.32, SD = 2.64.

### Experience of tick-borne disease in livestock

Across the livestock owner groups, 45% of the respondents had experience of diagnosed tick-borne diseases among their animals. This experience was more common among sheep owners (48%) and owners of sheep and cattle (51%) as compared to owners of cattle only (29%, *χ*^2^ = 17.68, *df* = 2, *P* < 0.001). Livestock owners that had experienced disease among their animals had done so for between less than one year up to 48 years, with an average of 10 years. Babesiosis was reported by 68% of cattle owners, 30% of owners of sheep + cattle and 6.4% of sheep owners. The symptoms mentioned were: reduced general condition, reduced milk production, emaciation, blood in urine, high fever, fatigue, joint inflammation and/or the animals had died. Anaplasmosis was reported by 38% of cattle owners, 76% of owners of sheep + cattle and 70% among sheep owners. The symptoms reported were reduced general condition, emaciation, blood in urine, high fever, fatigue, difficult breathing, inflamed lymph nodes, joint inflammation, limbing, unsteady walking, lame in hindleg and/or that animals had died.

### Appraisal dimensions of relevance and implications

**Fig. 2:**
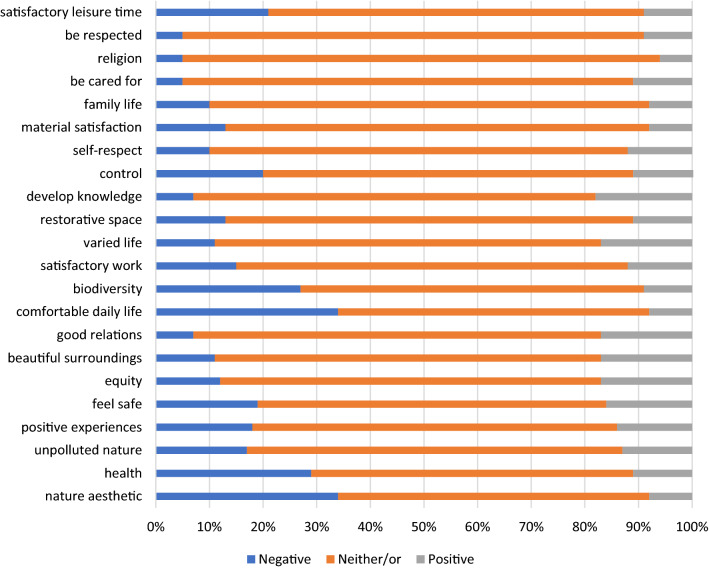
Livestock owners’ assessment of the impact of the tick-bites and tick-borne disease in livestock on different dimensions of their subjective quality of life in Norway

The effects of the presence of tick-borne disease in livestock was assessed to be of intermediate relevance to the livestock production in general (M = 2.58, SD = 1.15). The relevance significantly differed between the owner groups, *F*_(2, 772)_ = 5.07, *P* = 0.006, η_p_^2^ = 0.013. Bonferroni *post-hoc* test showed ‘relevance’ to be assessed significantly higher among sheep owners (M = 2.63, SD = 1.15) and owners of both cattle and sheep (M = 2.67, SD = 1.17) than among cattle owners (M = 2.31, SD = 1.05). However, this difference only explains a bit more than one percentage of the total variation in the answers and should not be given too much consideration. The presence of tick-borne diseases among livestock was not considered to have an effect of the 22 subjective quality of life scale, that is, approx. 70–80% of respondents checked the box for neither/or (Fig. 2). The subjective quality of life with regard to five domains addressed was, on average, considered to be slightly negatively affected. More than 20% of the respondents reported to be very negatively or somewhat negatively affected. These domains were: (i) have a good health; (ii) have a simple and comfortable everyday life; (iii) have a good quality of my spare time and be able to do things that I like; (iv) have access to nature environments with a plethora of plants and animals; (v) enjoy the beauty of nature and the cultural landscape. An overarching index for ‘implication’ was calculated by adding the responses for these five variables ranging from 1 (very negative implication) to 5 (very positive implication; Cronbach’s α = 0.89). The implications of the presence of tick-borne disease in livestock was assessed to be slightly negative (M = 2.78, SD = 0.60) regardless of livestock ownership, and there were no significant differences, *F*_(2, 772)_ = 1.84, *P* = 0.160.

### Coping potential, social trust and norm compatibility

The variables assessing coping potential were subject to an exploratory factor analysis with the objective to identify possible overarching coping strategies. Of the 15 items, 13 were satisfactorily normally distributed, i.e. the ratio of skewness and kurtosis, and its standard error, did not exceed 5. Two of the coping potential items were highly agreed on by almost everyone (consulted veterinarian, M = 4.54, SD = 0.76; used substances, for example treated the animals with a tick repellent, M = 4.73, SD = 0.64). These items did not meet the statistical requirements and were excluded from the factor analysis. A principal component analysis (PCA) was conducted on the remaining 13 items with orthogonal rotation (varimax) based on the requirements of eigenvalues above 1. Sampling adequacy was, with Kaiser-Mayer-Olkin index (KMO) = 0.81. Correlations between items were sufficiently high, as indicated by Bartlett’s test of spherity (*χ*^2^_(78)_ = 1843.94, *P* < 0.001). Three components explaining 50.58% of the variance were retained (Table 2). Inspection of the scree plot verified the number of components. The three components were reliable: Cronbach’s α for emotion-based coping was 0.70, for problem-solving based activity was 0.66 and for problem-solving based: information-seeking was 0.65. Three indices were calculated by averaging the included items with loadings 0.45 or above of each component for each participant. The item “avoid to talk about it” loading negatively on information-seeking was excluded from information-seeking since it substantially reduced the internal reliability of the index: emotion-based (M = 2.52, SD = 0.75, range: 1–5); problem-solving: action (M = 3.09, SD = 0.71, range: 1–5); and problem-solving information-seeking (M = 3.52, SD = 0.80, range: 1–5). Neither of the three coping strategies differed between the three livestock owner groups: emotion-based: *F*_(2, 772)_ = 0.95, *P* = 0.387; problem-solving: action: *F*_(2, 772)_ = 0.66, *P* = 0.518; and problem-solving information-seeking: *F*_(2, 772)_ = 0.77, *P* = 0.463. In the open-ended item on what strategies the livestock owners use, 76% reported that they today use some preventive actions to avoid that their animals get tick bites. Almost everyone stated that they systematically use tick repellents, but also other strategies were mentioned by a few respondents such as reducing encroachment and grooming the livestock. The use of preventive actions was significantly more frequently reported among sheep (81%) and both cattle and sheep (82%) than among cattle owners (54%), *χ*^2^ = 6.93, *df* = 2, *P* < 0.001.

The livestock owners expressed a slight social trust (M = 3.41, SD = 0.83). There was no difference between the three groups of livestock owners, *F*_(2, 772)_=1.72, *P* = 0.181. Thirty-five per cent of the respondents’ reported that they have received support in situations with tick-bites that resulted in illness. In the open-ended follow-up question most respondents reported this support to been given by other farmers, neighbors, and the veterinarian, a few mention NSG (stakeholder organization for sheep owners) and the county officer (Fylkesmannen, the regional state representative for the administration for animal management). This differed however significantly between the livestock owner groups cattle (25%), sheep (38%) and both (37%), *χ*^2^ = 6.93, *df* = 2, *P* = 0.030. About 10% reported that they had wished for support and help, including more knowledge from veterinarians and researchers, management of deer and information about preventive actions. This was most visible among the livestock owners with cattle and sheep (16%) as compared to sheep only (9%) and cattle only (5%), *χ*^2^ = 10.40, *df* = 2, *P* = 0.006.

The norm compatibility was in general high (M = 3.81, SD = 0.63), which most likely refer to the frequent use of tick repellents), but significantly differed between the owner groups (*F*_(2, 772)_ = 20.75, *P* < 0.001, η_p_^2^ = 0.051). Bonferroni *post-hoc* test showed norm compatibility to be significantly higher among sheep owners (M = 3.89, SD = 0.63) and owners of cattle + sheep (M = 3.84, SD = 0.62) as compared to cattle owners (M = 3.52, SD = 0.55).

### The contribution of emotional appraisals to worry and fear of tick-borne diseases

In a hierarchical multiple regression analysis with worry and fear of tick-borne diseases among livestock as the outcome variable, 35% of the variance could be explained. In the analysis livestock ownership was first introduced as a dummy variable. In the following steps, personal experience was added, followed by the emotional appraisal variables relevance, implication, social support and the three coping variables, and finally norm compatibility. Table 3 shows the first and the final model of the regression. In Model 1 current disease hazards could predict 3% of the variance in livestock owners’ worry and fear of tick-borne diseases among their livestock; after adding the psychological variables, the predicted variance substantially increased to 35%. More personal experience of tick-borne diseases among livestock, higher perceived relevance, negative implications and emotion-based coping stand out as the most important to experienced fear.

**Table 3:** Hierarchical multiple regression analysis with fear of tick-borne diseases among livestock as outcome variable

Fear of tick-borne disease	Model 1 Disease hazards (*n* = 704)			Final model Livestock owners’ appraisals (*n* = 704)		
	*B*	*SE B*	*β*	*B*	*SE B*	*β*
Constant	4.04	0.21		0.74	1.18	
Lyme borreliosis	4.32	2.22	0.07(*)	− 0.95	1.90	− 0.02
Anaplasmosis (sheep)	12.86	5.52	0.09	4.72	4.57	0.03
Anaplasmosis (cattle)	23.09	13.43	0.07*	16.28	11.04	0.05
Babesiosis	26.44	11.12	0.10*	− 0.37	9.34	− 0.01
Cattle = 1, Sheep + both = 2				0.08	0.29	0.01
Sheep = 1, Cattle + both = 2				− 0.06	0.22	− 0.01
Personal experience 1 = no, 2 = yes				0.97	0.22	0.17***
Relevance (low-high)				0.912	0.10	0.36***
Implication (negative-positive)				− 0.69	0.15	− 0.15***
Social trust (low-high)				− 0.04	0.11	− 0.01
Emotion based coping (low-high)				0.67	0.14	0.17***
Information-seeking coping (low-high)				0.30	0.13	0.08*
Action-based coping (low-high)				− 0.09	0.14	− 0.02
Norm compatibility (low-high)				− 0.07	0.15	− 0.01
	*F*_(4, 699)_ = 6.89, *P* < 0.001, *R*^2^ = .04, *R*^2adj^ = 0.03			*F*_(14, 689)_ = 28.03, *P* < 0.001, *R*^2^ = .36, *R*^2adj^ = 0.35		

*Notes*: In the first model, data for current disease hazards are entered as predictor variables. In the final model, the livestock owners’ emotional appraisals are added

(*)*P* = 0.052, *P < 0.05, **P < 0.01, ***P < 0.001

## Discussion

Tick-borne diseases in humans and livestock are emerging following expansion of tick ranges in many regions in Europe [1] and in North America [2]. People in the Nordic countries express worry and fear and take protective measures for themselves and their pets against tick-bites [23, 24]. Anaplasmosis and babesiosis constitute a real threat to the health of cattle and sheep, involving weight loss and increased mortality [31], but such disease emergences may have effects on livelihood and quality of life of farmers beyond this. Based on the theory of emotional appraisal, this study contributes with a nuanced understanding of livestock owners’ worry and fear of ticks and tickborne-diseases among their animals. The livestock owners who participated in this study in general expressed a moderate level of worry and fear of presence of ticks and tick-borne diseases in livestock. Despite that tick repellents, medication and land-use practice are available tools that to some extent can mitigate the negative effects of tick presence [32], approximately 30% of the respondents clearly reported feelings of worry and fear in the self-report scale. The emotional feelings the threat ticks pose should, therefore, not be neglected by authorities and stakeholder organizations.

### Disease levels, experience and appraisals

The association between estimated prevalence of tick-borne diseases in livestock and livestock owners’ worry and fear was weak. Rather, the livestock owners’ personal *previous experience* of ticks and tick-borne diseases in livestock as well as they *appraisals* i.e. interpretation of the situation, were associated with relatively stronger feelings of worry and fear. A substantial amount of the livestock owners (45%) had experience of diagnosed tick-borne diseases among their animals, and the livestock owners reported extensively on the experienced symptoms among their animals. When the probability of tick-borne diseases in livestock is present and the respondents have previous experience of such diseases, they are likely to account of earlier problems *via* associative networks of memories of the previous occurrence [33], and thus fearing a repetition of the situation [34]. A next step of the stimulus evaluation checks in the appraisal process relates to a high *perceived relevance* of tick-borne diseases in livestock to one’s farming practice. We found perceived *negative implications* of presence of ticks and tick-borne diseases in livestock to one’s quality of life contribute to explain the variation in worry and fear among the livestock owners. In line with the moderate level of worry and fear reported, the potential threat of ticks in general was reported to be of intermediate levels of relevance, again with a large variation between respondents. The perceived quality of life implications relates to a broad area of quality of life aspects, and if considered to be affected, these aspects were primarily considered to be negatively affected. This was most notable for aspects related to personal health, possibilities for a comfortable life, leisure time and recreational opportunities. These quality of life aspects could directly be associated to an increased workload/stress among the livestock owners. Moreover, quality of life aspects associated with the personal appreciation of the aesthetics of nature and benefits of biodiversity were perceived to be negatively affected. It might be that if the presence of ticks is associated with stress it may also alter the view of nature.

Coping potential refers to the livestock owner’s way of handling the stress. As for *coping potential*, three different strategies could be identified: one emotion-focused coping strategy and two problem-solving-based coping strategies (information-seeking and action-based). Handling the situation by becoming emotional, i.e. employing emotion-focused coping strategies, about the situation and/or relying on information seeking are associated with relatively higher worry and fear. The emotion-based coping strategy includes for example that the livestock owner avoids talking about the problems experienced and becomes afraid of being regarded as a lousy animal keeper, which seems to increase worries and fear. As emotion-based coping is partly related to not talking about the problems with ticks and the tick-borne diseases in livestock, there is also risk for further decrease in the livestock owner’s psychological wellbeing. This implies that it is important for veterinarians, representatives of authorities and stakeholder organizations to actively address livestock owners’ feelings in the discussion about ticks. Information-seeking coping can be interpreted as an attempt to get more action possibilities to handle the situation, if or when known coping strategies may not work as most livestock owners in the investigated area already use tick repellents. The association between information-seeking and relatively higher level of worry and fear stresses the need of supporting livestock owners in translating potential action-based coping strategies into manifest actions. This can be compared with strengthening self-efficacy, in previous research pointed at as critical to adopt protective behavior to tackle the risks for humans [1, 16]. Action-based coping included changes in land use practice and production. These strategies seem to be used by the livestock owners regardless of their level of worry and fear, and may be more dependent on access to land, financial and labour-related resources [35].

Social support in terms of being given the possibility to express the feelings (i.e. a form of emotional disclosure [36]), may facilitate coping. The livestock owners commonly reported that they handle tick-borne diseases in livestock with support from colleagues and neighbours, contact with a veterinarian and, as above mentioned, use of repellents. This becomes especially important for those farmers that responded with agreeing on the item “avoid talking about it”. To have a social network thereby seems to be a key component. In previous research on fear of large carnivores, social trust in managing authorities has been shown to reduce such negative feelings [29, 37]. In the case of ticks there seems to be little controversy, why social trust may be of less relevance. Also, there seems to be a unitary norm around the problem and how it should be tackled, this is most likely why norm compatibility is not significantly associated with worry and fear.

### Comparing livestock owners

This study included livestock owners with cattle and/or sheep. Despite differences in farming practice, the livestock owners reported similar levels of worry and fear. Sheep owners to a larger extent had previous experiences of tick-borne diseases among their animals than did the cattle owners. Also, sheep owners perceived the threat of tick-borne diseases in livestock to be more relevant to their situation. The perceived implications of disease and coping potential was however very similar across the livestock owner groups. Current norms around prevention and treatment of livestock seems to be somewhat more congruent in the case of sheep than in the case of cattle. In sum, although sheep owners have more negative experiences and find the situation more relevant, the cattle owner face less congruent norms around how they could or should cope with the situation, leaving the different livestock owners in about the same position when it comes to the stressfulness in the event of tick-borne diseases in livestock.

The results are based on self-report data among members of two large livestock organizations in Norway (TINE and NSG). Data were collected by a web-based questionnaire and among members of NSG the online version was complemented by a paper and pencil version. The number of reminders were limited by the organizations willingness to send out repeated e-mails to their member lists. The response rate was 27%, which puts limitations on the possibilities to generalize the results but is likely to be sufficient to give an understanding of the pattern of the livestock owners’ appraisal process. It is likely that livestock owners who found the threat of ticks and tick-borne diseases in livestock to be more relevant to be more prone to answer, comparing the response rate between cattle and sheep owners, it might also be that the paper version used by NSG was more suitable for this group of respondents.

## Conclusions

Taken together, the results show that the stimulus evaluation checks of emotional appraisal theory [21] can be used as a basis to understand the process behind livestock owners’ worry and fear. The study thereby strongly supports the need to integrate psychology in biodiversity conservation [38] and the use of behavioral science theories to understand human responses to increasing presence of ticks in the environment [13]. Focusing upon the livestock owners’ personal experiences and their own interpretation of the current situation in Norway, this study stresses the importance of understanding the livestock owners’ perceived personal relevance of the presence of ticks, its potential negative implications for the livestock owners’ daily life at large, and what potential the livestock owner has to cope by different strategies to adapt or adjust to the situation. The results point to the need of shifting focus from a singular use of data of estimated hazards of tick-borne diseases to livestock towards a more holistic approach where the livestock owner is in the center.

## Data Availability

Data supporting the conclusions of this article are included within the article. The datasets used and/or analysed during the present study are available from the corresponding author upon reasonable request.

## Abbreviations

ANOVA: analysis of variance
M: mean value
n: sample size
NSG: Norsk Sau og Geit, stakeholder organisation (sheep)
PCA: principal components analysis
SD: standard deviation
TBE: tick-borne encephalitis
TINE: stakeholder organization (cattle)
η_p_^2^: partial eta-squared
